# Slovenian Pet Owners' Experience, Attitudes, and Predictors Regarding Cannabinoid Use in Dogs and Cats

**DOI:** 10.3389/fvets.2021.796673

**Published:** 2022-01-05

**Authors:** Katerina Tomsič, Kristina Rakinić, Alenka Seliškar

**Affiliations:** ^1^Veterinary Faculty, University of Ljubljana, Ljubljana, Slovenia; ^2^Faculty of Social Sciences, University of Ljubljana, Ljubljana, Slovenia

**Keywords:** cannabinoid, attitudes, experience, predictors, dogs, cats, survey, postmodern health values

## Abstract

The aim of this study was to assess the personal experience and attitudes of Slovenian pet owners regarding cannabinoid (CBD) use and to identify the predictors of the first use and reuse of CBDs in dogs and cats. We hypothesized that positive attitudes toward CBDs, postmodern health values, and personal experience would be significant predictors of CBD use in animals. An open online survey targeted randomly selected Slovenian dog and cat owners, regardless of their experience with cannabis products. The questionnaire consisted of six sections related to demographic data and personal experience with CBD use, information about the participant's animal, experience with CBD use in the participant's animal, reasons for not using CBDs in their animal, attitudes toward CBD use in dogs and cats, and postmodern health values. Descriptive statistics were performed to analyze demographics, personal experience with CBD use, and experience with CBD use in dogs and cats. Hierarchical multiple regression using the enter method was performed to analyze the important predictors of CBD use. A total of 408 completed questionnaires were included in the statistical analysis. A substantial proportion (38.5%) of owners had already used CBDs to treat their animal. Positive attitudes and previous personal experience were significant (*p* < *0.05)* predictors of first use and reuse of CBDs in pets, while postmodern health values were not. In conclusion, the decision to use CBDs for medicinal purposes is based on acquired information and personal experience. Veterinarians should be informed and familiar with CBDs as a treatment option. However, further research is essential to establish the use of CBDs in veterinary medicine. Improved laws and regulations are also needed to ensure that only high-quality medications are prescribed to dogs and cats.

## Introduction

At every time and place in human history, there are prevailing values that guide people's ideas of what is right and good. Nowadays, the main organizing principles of postmodern society are consumption rather than production (1) and the ability to choose from a variety of options in contemporary Western society (2). According to Bakx (3), there have been three significant cultural shifts in the transition from late modernity to postmodernity: the rejection of authority (especially scientific authority), an increase in consumerism, and the importance of individual responsibility for health. These shifts in values may explain the increasing popularity of complementary and alternative medicine (CAM) (4). Six main health values are evident in postmodern society: nature and natural remedies (appreciation of natural foods, avoidance of artificial ingredients), anti-science attitudes, holism (integration and balance of body, mind, and spirit), rejection of authority, individual responsibility, and consumerism. The value of individual responsibility is an important belief that characterizes the postmodern age, as people tend to believe that they are responsible for every aspect of their lives, including their health (5). Postmodern health values have been reported as a significant predictor of the actual use of and positive attitudes toward CAM (6).

Cannabinoids (CBDs) are recognized as a CAM treatment. They are active chemical substances that bind to the receptors of the endocannabinoid system in the body and exert different effects. The best-known CBDs are the psychoactive compound delta-9-tetrahydrocannabinol (THC) and the non-psychoactive cannabinoid cannabidiol, which is thought to exert many beneficial effects (7).

Phytocannabinoids are CBDs that occur naturally in the cannabis plant, which has been used as an industrial material throughout human history and has also been included in pharmacopeias as a cure for many diseases (8, 9). In the twentieth century, the criminalization of cannabis took place worldwide, including in European countries (10, 11). In the early 1990s, new laws were enacted allowing cannabis use under strict conditions, including the use of cannabis-based medicines, partly due to renewed scientific interest triggered by anecdotal reports of the medicinal use of cannabis-based products (10, 12). Despite legal restrictions on the use of cannabis products, there is growing evidence that people are using them to treat conditions that cannot be cured with conventional medications or to support current treatments (13–18). In addition, the use of such products is extended to family members, including children (19, 20) and even pets (21–23).

In Slovenia, the use of medical cannabis is allowed (24), but there are still some medical and legal controversies that prevent physicians from prescribing cannabis-based medicines (25), along with the stigmatization of cannabis users (8, 26). Cannabinoid food products for dogs and cats are classified as novel foods (27) and are marketed as dietary supplements for animals.

This study set out to obtain information on the experience and attitudes of Slovenian pet owners with respect to CBD use and to identify predictors of first use and reuse of CBDs in dogs and cats. We hypothesized that positive attitudes toward CBDs, postmodern health values, and previous experience would be significant predictors of CBD use in animals.

## Materials and Methods

### Preliminary Study

A preliminary study was conducted in 106 dog and cat owners. The aim was to test the psychometric properties of the two scales and to investigate the meaningfulness of the questions. The questionnaire was distributed in paper-pencil form to clients of the Small Animal Clinic (Veterinary Faculty, University of Ljubljana), clinic staff, members of various canine associations, and acquaintances of the researchers. A convenience sample was obtained by the snowball method (the first participants were asked to distribute the questionnaire among the pet owners they knew). The comprehensibility of the questions was first tested by the researchers. Some of the questions were excluded as the researchers felt that they were not relevant to the aim of the study. The scale developed by the researchers to assess dog and cat owners' attitudes toward the use of CBDs showed good psychometric properties and was therefore used in the main study. The scale, which assessed postmodern health values, required some modifications, which are described below. Since the research was conducted during the second dissemination wave of COVID-19 in winter 2020–2021, the researchers decided that it would be safer and more ethical to conduct it online.

### Questionnaire Design of the Main Study

The questionnaire was created using the open-source survey application 1ka (www.1ka.si) and targeted dog and cat owners, regardless of their experience with cannabis products. Participants were selected by convenience sampling. The link to the questionnaire was distributed through the researcher's circle of acquaintances. In addition, various animal protection societies were contacted and then shared the link to the questionnaire through their Facebook websites. The link to the questionnaire was also published on the website and Facebook page of the Small Animal Clinic. Participation in the survey was voluntary, and no reward was offered for completing the questionnaire.

The aim of the survey, information about the researchers, a brief definition of CBDs, a declaration of anonymity, and the approximate time needed to complete the survey (5 mins) were provided at the beginning of the questionnaire. No personal data were collected or stored. The usability and functionality of the questionnaire were tested by the researchers before fielding. The open survey was advertised on the website of the Small Animal Clinic of the Veterinary Faculty, University of Ljubljana (www.kmz.si) and on the Facebook page of the clinic. A link to the survey was also shared among the researchers' acquaintances and spread using the snowball method.

The questionnaire consisted of 21 questions divided into six sections pertaining to demographic data and personal experience with CBD use, information about the participant's animal, experience with CBD use in the participant's animal, reasons for not using CBDs in the animal, attitudes toward CBD use in dogs and cats, and postmodern health values. Adaptive questioning was used to allow participants who had had no experience with CBDs to skip the questions related to experience with their use. The questionnaire could only be submitted if mandatory questions were answered, and the questionnaire could be reviewed before completion using a back button at the bottom of each page. The online questionnaire was available from 27 November 2020 to 11 February 2021.

### Demographic Variables and Experience With Personal use of CBDs

The first part of the questionnaire included demographic variables (gender, age, education level) and a question related to previous experience with CBDs (Have you ever used CBDs to treat your medical condition?). If the answer was positive, the participant had to rate the effectiveness of CBDs on a 5-point Likert scale (1—completely ineffective, 5—very effective) and the likelihood of using CBDs again in the future (1—not at all likely, 5—very likely). A negative response required the participant to rate on a 5-point Likert scale the likelihood of using CBDs in the future (1—not at all likely, 5—very likely).

### Animal Data

Questions about the participant's animal included the species (dog or cat) and the animal's health status, as well as whether the participant had already used CBDs to treat his/her animal in the past (yes/no).

### Experience With the use of CBDs in Animals

Participants who answered in the affirmative to the question about the use of CBDs in pets were directed to this section. Participants were asked to (a) rate the effectiveness of CBDs on a 5-point Likert scale (1—completely ineffective, 5—very effective); (b) indicate sources of information about CBDs (veterinarians, internet, acquaintances, pet stores, others); (c) indicate positive effects (greater liveliness, improved well-being, improved appetite, improved mobility), adverse effects (fatigue, excessive appetite/thirst, dizziness), or no effect; (d) indicate whether they would use CBDs in the future on a 5-point Likert scale (1—very unlikely, 5—very likely); (e) report whether treatment with CBDs was their first choice, the choice when all other medications failed, or used as supportive therapy; f) indicate the duration of CBD use (<1 month, <6 months, more than six months).

### Reasons for Not Using CBDs in Animals

Participants who answered negatively to the question about the use of CBDs in pets were referred to this section. They were asked to select reasons for not using CBDs from a list: I have not yet considered this option; my animal has not had any health problems that could be alleviated or treated with CBDs; lack of information on where to buy CBDs; safety concerns; legal reservations; lack of scientific evidence on their efficacy; lack of standardized/controlled products on the market; other. The next question required owners to indicate on a five-point Likert scale (1—very unlikely, 5—very likely) how likely they were to use CBDs in the future.

### Attitudes Toward the use of CBDs in Dogs and Cats

This section consisted of eight items (Table 1), and participants responded on a 5-point Likert scale (1—strongly disagree, 5—strongly agree). Four items were negatively worded and were reversed for analysis. Responses were then averaged, with higher scores indicating more positive attitudes. The scale was created by the authors and tested in a preliminary study with an internal reliability coefficient α = 0.82. Internal reliability was good (α = 0.85).

**Table 1:** Descriptive statistics for each of the items and total score of the short scale of attitudes toward cannabinoids (CBDs)^a^.

**Item**	**M ± SD^***b***^**	**Min**	**Max**
CBDs are effective medicine for treatment of various diseases in dogs and cats.	3.86 ± 0.88	1	5
Cannabinoid use should not violate legislation when treating dogs and cats.	4.45 ± 0.84	1	5
CBDs are natural remedies and so they are more suitable for treatment of dogs and cats as synthetic medicines.	3.62 ± 1.23	1	5
CBDs are only currently popular in the treatment of dogs and cats.*	4.10 ± 1.04	1	5
There is not enough scientific evidence supporting the efficacy of CBDs for treatment of dogs and cats to be able to trust them.*	3.45 ± 1.1 3	1	5
I would recommend to my friends the use of CBDs for the treatment of their animals.	3.78 ± 1.09	1	5
CBDs can cause animals more harm than good.*	4.07 ± 1.02	1	5
I would refuse CBDs as a medicine for my animal, even if their used was advised to me by a veterinarian.*	4.66 ± 0.72	1	5
**Total score**	**4.00** **±** **0.70**	**2**	**5**

*^a^Ranking performed on a scale where 1 = strongly disagree and 5 = strongly agree.*

*^b^Mean with standard deviation.*

*Reverse items.

### Postmodern Values About Health

In the preliminary study, postmodern values about health were measured using a 4-point scale (1—strongly disagree, 4—strongly agree) developed by Siahpush (5). The six postmodern values measured were natural remedies (item example: “I prefer natural remedies to chemical drugs.”), anti-science sentiment (item example: “Technological advances create an environment that is harmful to people.”), holism (item example: “I think my body has a natural ability to heal itself.”), rejection of authority (item example: “Patients should be able to have an input in what remedies health practitioners prescribe.”), individual responsibility (item example: “It is ultimately the individual who is responsible for his/her health.”), and consumerist attitudes toward health care (item example: “It's good that nowadays we have so many different types of therapies to choose from.”). The investigators obtained permission from the author to translate the questionnaire into Slovenian for use in this study. The questionnaire was translated using back-translation. The internal reliability of the scale ranged from α = 0.51 to 0.76 in the preliminary study. In the online questionnaire, the investigators modified the response scale by using a 7-point Likert scale (1—strongly disagree, 7—strongly agree), which allowed participants to give a neutral response (value 4). Two items representing the value of anti-science sentiment were excluded because they were not positively correlated in the preliminary study. In the online survey, the internal reliability for the other four scales ranged from α = 0.64 to 0.74, with a higher overall value representing a higher expression of postmodern health values. The internal reliability of the entire questionnaire was α = 0.85.

### Statistical Analysis

The authors set the minimum number of completed questionnaires at 384. The calculation was based on data from 250,000 registered dogs in Slovenia and aimed for a 95% confidence level to achieve actual values within ± 5% of the measured values.

Descriptive statistics were performed to analyze demographic data, personal experience with CBD use, and experience with CBD use in dogs or cats. The data are presented as mean ± standard deviation or percentage. A Cohen′s Kappa was used for comparison between own and animal use of CBD. Hierarchical multiple regression using the enter method was performed to analyze the important predictors of first use and reuse of CBDs. The data were analyzed using SPSS version 22 and RStudio version 1.3.1093. The level of significance was set at *p* < *0.05*.

## Results

A total of 408 owners answered all the questions, and only they were included in the final analysis. There were a total of 1,027 clicks on the survey link. Out of 554 persons who clicked on the survey start, 500 completed the first page of the survey, for a participation rate of 90%. Of the 500 participants who completed the first page, 412 also completed the last page of the survey, for a completion rate of 82.74%. For the final analysis, four participants were excluded due to missing data.

### Demographic and Animal Data

The majority of participants (85.8%) were female. The mean age was 39.2 years ± 11.6 years. Educational levels attained were, in descending order: master's degree 32.6%, high school 29.7%, bachelor's degree 14.5%, doctorate 7.4%, post-secondary vocational school 6.6%, vocational high school 4.4%, Master of Science 3.2%, and elementary school 1.7%.

The majority of participants were dog owners (76.7%). More than half of the owners (63.5%) reported that their animal was healthy. A substantial proportion of owners (42.2%) had already used CBDs to treat themselves. They found CBDs effective (3.97 ± 0.99) and were very likely to use them again (4.34 ± 1.02). Those who had not used CBDs expressed a relatively strong intention to use them in the future on a 5-point probability scale (3.34 ± 1.19).

### Experience of Owners Who Had Already Used CBDs in Their Animals

A substantial proportion (38.5%) of owners had already used CBDs in their animals. Most owners obtained information about CBD use in animals online (45.2%), followed by information from acquaintances (40.8%) and veterinarians (34.4%); 12.1 % of owners stated that they received the information elsewhere, mostly from manufacturers, at the Veterinary Faculty, from a dog therapist, at a dog show, etc.; and 3.2 % of owners received information about cannabis products at a pet store.

Table 2 shows the percentage of positive and adverse effects that owners attributed to CBDs in their animal. In general, positive effects predominated. The largest percentage of owners reported the improved well-being of their pet. Other positive effects reported by participants were calmness, relaxation, absence of fear of fireworks, reduction in the number of tumors, faster wound healing, thicker hair, etc. Other adverse effects reported were drowsiness, dry eyes, and vomiting; however, more than half of the participants reported none of these effects.

**Table 2:** The percentage of positive and adverse effects of cannabinoids.

**Positive effects**	%*
Improved well-being	72.0
Greater liveliness	35.7
Improved mobility	34.4
Other	29.9
Improved appetite	28.0
No effect	8.3
**Adverse effects**	**%**
No effect	57.3
Other	26.1
Dizziness	10.8
Excessive appetite/thirst	8.3
Fatigue	6.4

*Owners were able to select more than one category.

Over half (51.0%) of participants reported using CBDs as supportive therapy in addition to the conventional treatment; 22.9% indicated that CBDs were their first choice intended as the only therapy; 19.7% used CBDs because of failure of previous treatment; and 6.4% of owners responded with “other.” Of the owners using CBDs to treat their animals, 43.9% had been using CBDs for more than half a year, 35.7% for less than half a year, and 20.4% for less than a month.

Overall, owners considered CBDs effective in treating or alleviating their animals' health issues (3.99 ± 1.07) and reported a strong intention to use CBDs again in the future (4.31 ± 1.10).

### Owners Who Had Not Used CBDs in Their Animals

The majority (61.5%) of participants had not used CBDs to treat their animals. The main reason was that owners felt that their animals' health issues could not be alleviated or treated with CBDs (72.5%). Other reasons included the following: owners had not yet considered this option (31.1%); lack of information on where CBDs could be purchased (19.9%); lack of standardized and controlled products marketed in Slovenia (15.1%); concerns about the safety of CBD use (12.7%); and concerns about lack of scientific evidence on their efficacy (12.0%). A small percentage (3.2%) of owners indicated that they had legal concerns, about the same percentage that answered “other.” Overall, participants were inclined to use CBDs to treat their animals in the future (3.49 ± 1.19).

### Attitudes Toward the Use of CBDs to Treat the Animals and Predictors of (Potential) Use and Postmodern Health Values

Descriptive statistics for individual items and total average score on the short scale for attitudes toward the use of CBDs in dogs and cats are summarized in Table 1. Participants (*N* = 408) showed an overall positive attitude toward the use of CBDs in dogs and cats. Descriptive statistics for subscales and the total scale of postmodern health values are summarized in Table 3. Participants (*N* = 408) showed an overall high expression of importance of postmodern health values. They particularly valued a diversity of different health treatment options (presenting value of consumerism), and they found it important that each individual is responsible for his or her own health (representing the value of individual responsibility).

**Table 3:** Descriptive statistics for postmodern health values^a^.

**Subscales**	**M ± SD^**b**^**	**Min**	**Max**
Nature and natural remedies	4.68 ± 1.17	1.40	7.00
Holism	5.49 ± 0.89	2.40	7.00
Rejection of authority	5.33 ± 1.07	2.20	7.00
Individual responsibility	5.76 ± 0.91	1.50	7.00
Consumerism	6.25 ± 1.12	1.00	7.00
**Total scale**	**5.34** **±** **0.74**	**3.25**	**7.00**

*^a^Ranking performed on a scale where 1 = strongly disagree and 7 = strongly agree.*

*^b^Mean with standard deviation*.

### Predictors of the use of CBDs in Dogs and Cats

Comparison of the personal use of CBDs and the use of CBDs in animals is shown in Table 4. There was slight agreement between these two types of CBDs use, K =0.18, *p* < 0.001.

**Table 4:** Relationship between the owner's personal cannabinoid (CBD) use and use in dogs and cats.

		**Personal use of CBDs**		
		**NO**	**YES**	**TOTAL**
Use of CBDs in the animal	NO	186 (79%)	65 (38%)	251 (62%)
	YES	50 (21%)	107 (62%)	157 (38%)
	TOTAL	236 (58%)	172 (42%)	408 (100%)

Hierarchical multiple regression (enter method) was performed to determine the predictors of the likelihood of the first use of CBDs in animals. Owners with no experience with CBD use in their animals were included (*N* = 251). Model 1 (Table 5) indicated that lower education was a significant predictor of the likelihood of first use of CBDs to treat animals. Model 1 was not significantly better than Model 0, with Model 1 explaining only 2% of the total variance. In the second step, attitudes toward CBDs and postmodern health values were added. The only significant predictor of future CBD use in pets was a positive attitude toward CBDs (*p* < 0.05). Model 2 explained 49% of the total variance and was significantly better than Model 1 (*p* < 0.001).

**Table 5:** Hierarchical multiple regression for predicting the first use of cannabinoids (CBDs).

	**Likelihood of the first use of CBDs**				
		**Model 1**		**Model 2**	
		***B [95% CI]***	**β**	***B [95% CI]***	**β**
Step 1:	Constant	3.02 [2.05, 3.98]		−1.24 [−2.27, −0.21]	
	Gender	0.26 [−0.15, 0.66]	0.08	0.05 [−0.24, 0.34]	0.02
	Age	0.01 [−0.00, 0.02]	0.11	0.00 [−0.01, 0.01]	0.03
	Education level	−0.09 [−0.17, −0.01]	−0.13*	0.00 [−0.06, 0.07]	0.00
Step 2:	Attitudes toward CBDs			1.27 [1.08, 1.45]	0.72**
	Postmodern health values			−0.5 [−0.22, 0.12]	−0.03
	*ΔR^2^*	0.03		0.47**	
	*Adj. R^2^*	0.02		0.49**	
	*F for change in R^2^*	2.55		114.38	

*CI = confidence interval. The coding for gender: 0—female, 1—male.*

*B is the rate of change per unit. β is the correlation coefficient ranging from 0 to ±1.*

*R^2^ is a statistical measure that indicates the proportion of the variance of a dependent variable that is explained by one or more independent variables. ΔR^2^ is the change in R-square when the predictors of the second model are added. Adjusted R-squared adjusts the statistic based on the number of independent variables in the model.*

*The F-statistic compares a model with zero predictor variables or the previous model and decides whether the added coefficients improved the model.*

*p < 0.05, **p < 0.001.

Hierarchical multiple regression (method enter) was performed to assess which of the predictors influence the likelihood of reuse of CBDs in the future (Table 6). Owners with experience of using CBDs in pets were included (*N* = 157). In Model 1 demographic variables were included. There were no significant predictors, and the model explained 0% of the total variance. In the second step the evaluation of the efficacy of CBDs was included, which turned out to be a strong predictor of its reuse (*p* < 0.001), and Model 2 explained 60% of the total variance. In the third step, attitudes and values were included. The efficacy of CBDs remained a significant predictor, but a positive attitude was also recognized as a significant predictor of the more likely reuse of CBDs in pets in the future. Model 3 was significantly better than Model 2 and explained 68% of the total variance.

**Table 6:** Hierarchical multiple regression for predicting re-use of cannabinoids (CBDs).

		**Likelihood of CBD re-use**					
		**Model 1**		**Model 2**		**Model 3**	
		***B [95% CI]***	**β**	***B [95% CI]***	**β**	***B [95% CI]***	**β**
Step 1:	Constant	4.76 [3.51, 6.01]		0.56 [−0.40, 1.51]		−0.69 [−1.84, 0.45]	
	Gender	−0.07 [−0.64, 0.49]	−0.02	0.06 [−0.29, 0.42]	0.02	−0.06 [−0.39, 0.26]	−0.02
	Age	0.00 [−0.02, 0.02]	−0.00	0.01 [−0.00, 0.02]	0.09	0.01 [−0.00, 0.02]	0.06
	Education level	0.07 [−0.17, 0.04]	−0.01	0.01 [−0.06, 0.08]	0.01	0.03 [−0.03, 0.09]	0.05
Step 2:	Efficacy of CBDs			0.82 [0.71, 0.92]	0.79*	0.58 [0.45, 0,70]	0.59*
Step 3:	Attitudes toward CBDs					0.76 [0.52, 1.01]	0.41*
	Postmodern health values					−0.16 [−0.32, 0.01]	−0.10
	*ΔR^2^*	0.01		0.61*		0.08*	
	*Adj. R^2^*	−0.01	0.60*			0.68*	
	*F for change in R^2^*	0.59	233.91			18.82	

*CI = confidence interval. The coding for gender: 0—female, 1—male.*

*B is the rate of change per unit. β is the correlation coefficient ranging from 0 to ±1.*

*R^2^ is a statistical measure that indicates the proportion of the variance of a dependent variable that is explained by one or more independent variables. ΔR^2^ is the change in R-square when the predictors of the second model are added. Adjusted R-squared adjusts the statistic based on the number of independent variables in the model.*

*The F-statistic compares a model with zero predictor variables or the previous model and decides whether the added coefficients improved the model.*

*p < 0.001.

## Discussion

The experience and attitudes of pet owners with respect to CBD use in their pets have been studied in the USA (22) and Canada (23), but not in Europe. Dog and cat owners who participated in this study generally had a positive experience and positive attitudes with respect to the use of CBDs in their pets. Positive attitudes and previous personal experience with CBDs, but not postmodern health values, were significant predictors of CBD use in their animals.

People rely on cannabis products to treat many diagnosable conditions (28–30), and self-treatment was also reported by 42.2% of our participants. The overall experience of personal CBD use was positive, as reported in other studies (28–30), and our participants were very likely to use CBDs again in the future. Participants who did not use CBDs for themselves expressed a relatively strong intention to use them in the future. In fact, cannabinoid products are widely accepted by consumers as a treatment for certain health conditions as well as to maintain general health and well-being (28). The use of medical cannabis is generally accepted by physicians as a valid treatment option for cancer, muscle spasms, seizures, and glaucoma, as well as for the alleviation of symptoms such as pain, nausea/vomiting, and anxiety (31, 32).

The main source of information about cannabinoid products in our study was the internet, followed by advice from acquaintances, which is consistent with previous studies (21, 28). In addition, veterinarians were a strong source of information in our survey. The rapidly growing market for cannabis-based products and the widespread acceptance of these products as an effective treatment for pets have challenged veterinarians to gather as much scientific data as possible on the use of CBDs (21–23). Moreover, emerging scientific evidence supports the use of CBDs in dogs and cats (33–40).

The participants in our study predominantly reported positive effects of CBDs in their animals, which is broadly consistent with previous studies (21, 23). The positive effects observed in our study (improved well-being, greater liveliness, increased activity) were also reported in other studies, in which improved well-being, increased activity, and pain reduction were the most common positive effects (21, 23). The adverse effects, such as sedation and/or drowsiness and increased appetite and thirst, reported in our study were also observed by pet owners in other studies (38, 39). However, the effects reported in this study are the subjective evaluation of dog and cat owners and should not be interpreted as an evidence-based finding.

The dog and cat owners in our survey used CBD products in their pets mainly as supportive therapy. On the other hand, there were participants who resorted to CBDs because conventional treatment failed, and some of them used CBD products as the only remedy. Among the dog and cat owners who did not use CBDs in their animals, the main reasons cited for not using them were that there was no need to use them (healthy animal) or there was a lack of information about the use of CBDs in dogs and cats. Kogan et al. (23) came to similar conclusions in a survey study of Canadian dog owners' use and perceptions of cannabis products.

Overall, owners showed a positive attitude toward the use of CBDs in their animals. They strongly agreed that it should not conflict with legislation, and they would not refuse the use of CBDs as a medicine for their animal if it was advised by their veterinarian. Participants agreed that CBDs are an effective treatment and are better suited as a natural remedy for treating animals than synthetic medications. This statement reflects the postmodern appreciation of nature and natural remedies described by Shiapush (5). In O'Callaghan & Jordan's study (6) of postmodern values, attitudes, and the use of CAM, postmodern values overall were associated with positive attitudes toward CAM, although only natural remedies and rejection of authority were significant predictors, which is in partial agreement with our findings. In our study consumerism and individual responsibility were the most expressed postmodern values, which is in agreement with the main organizing principles of postmodern society (1–3). However, postmodern values were not identified as predictors of CBD use in dogs and cats.

A slight association between the personal use of CBDs and use of CBDs in animals was observed in our study. Owners tended to do what they believed was best for their animals, based on their own experience. Accordingly, if owners had a positive experience or any experience with CBDs, they were more likely to use them in their animals. As observed by Kogan et al. (22), dog owners who were using medical marijuana products for themselves were more likely to purchase similar products for their dogs.

The only significant predictor of the first use of CBDs in animals was a positive attitude toward CBDs, which explained nearly half of the variance in owners' intention to use CBDs in the future. Many studies have pointed to the importance of positive attitudes toward a particular object and future intention to use it (41–44). When evaluating predictors of reuse of CBDs in animals, we found that CBD efficacy ratings and positive attitudes were strong predictors of CBD reuse. It is well known that higher ratings of efficacy of certain products lead to the more likely use of these products in the future (13, 44). Contrary to our hypotheses, postmodern health values were not significant predictors of CBD first use and reuse in cats and dogs. As Coulter and Willis (4) noted regarding the postmodern thesis on the use of CAM, it is difficult to extrapolate causal relationships from cross-sectional survey data. More than 20 years ago, the social and medical views on alternative medicine in Slovenia were studied. The study revealed that people are less willing to accept conventional medicine as the only health option and rather rely on their social network to decide what, where and when to seek official or unofficial medical help (45). Our study confirmed that dog and cat owners in Slovenia rely on the internet and advice from acquaintances to seek information about CBD products.

Demographic variables were not significant predictors of the future use and reuse of CBDs in dogs and cats in this survey. This is in general agreement with the findings of Kogan et al. (22), who reported that the gender of dog owners was not correlated with the decision to purchase CBDs for the treatment of their dogs. In contrast to our findings, other studies reported that users of CAM tended to be younger, better educated, wealthier, of poorer health status, and female (6, 46, 47).

The authors would like to address some of the limitations of this study. First, the calculation of the power of the study was based on the number of dogs registered in Slovenia. Registration of cats is not mandatory, and their number is currently unavailable. Second, the convenience sample limits the generalization of the results to the Slovenian population and the results may be biased depending on the individuals who chose to participate in the survey. Third, the postmodern health values scale has some deficiencies with respect to its construction and structural validity, and in our study, some subscales showed low internal validity.

In conclusion, positive attitudes toward and personal experience with CBD use were the strongest predictors for CBD first use and reuse in dogs and cats, whereas postmodern health values were not. The use of cannabis-based products is increasing worldwide as well as among pet owners in Slovenia. Conventional medicine and scientific research should aim to evaluate and include CBDs as a treatment option in dogs and cats. Improved laws and regulations are also needed to ensure that only high-quality medications are prescribed to dogs and cats.

## Data Availability Statement

The raw data supporting the conclusions of this article will be made available by the authors, without undue reservation.

## Author Contributions

KR performed the statistical analysis. KR and KT drafted the manuscript. AS revised the draft manuscript. All authors contributed to the design of the study, including the creation and implementation of the questionnaire, and approved the submitted version of the manuscript.

## Funding

This study was supported by the Slovenian Research Agency (P4-0053).

## Conflict of Interest

The authors declare that the research was conducted in the absence of any commercial or financial relationships that could be construed as a potential conflict of interest.

## Publisher's Note

All claims expressed in this article are solely those of the authors and do not necessarily represent those of their affiliated organizations, or those of the publisher, the editors and the reviewers. Any product that may be evaluated in this article, or claim that may be made by its manufacturer, is not guaranteed or endorsed by the publisher.

## References

[1] Giddens A. Modernity and Self-identity: Self and Society in the Late Modern age. London: Stanford University press. (1991), p. 2164.

[2] Rayner L Easthope G. Postmodern consumption and alternative medications. J Sociol. (2001) 37:157–76. 10.1177/144078301128756274

[3] Bakx K. The eclipse of folk medicine in western society. Soc Health Illness. (1991) 13:20–38. 10.1111/1467-9566.ep11340307

[4] Coulter ID Willis EM. The rise and rise of complementary and alternative medicine: a sociological perspective. Med J Aust. (2004) 180:587–9. 10.5694/j.1326-5377.2004.tb06099.x 15174992

[5] Siahpush M. Postmodern attitudes about health: a population-based exploratory study. Complement Ther Med. (1999) 7:164–9. 10.1016/S0965-2299(99)80124-1 10581826

[6] O'Callaghan FV Jordan N. Postmodern values, attitudes and the use of complementary medicine. Complement Ther Med. (2003) 11:28–32. 10.1016/S0965-2299(02)00109-7 12667972

[7] Cristino L Bisogno T Di Marzo V. Cannabinoids and the expanded endocannabinoid system in neurological disorders. Nat Rev Neurol. (2020) 16:9–29. 10.1038/s41582-019-0284-z 31831863

[8] Bridgeman MB Abazia DT. Medicinal cannabis: history, pharmacology, and implications for the acute care setting. P T. (2017) 42:180–8. 28250701 PMC5312634

[9] Pisanti S Bifulco M. Modern history of medical cannabis: from widespread use to prohibitionism and back. Trends Pharmacol Sci. (2017) 38:195–8. 10.1016/j.tips.2016.12.002 28095988

[10] Zuardi AW. History of cannabis as a medicine: a review. Rev Bras Psiquiatr. (2006) 28:153–7. 10.1590/S1516-44462006000200015 16810401

[11] Mead A. Legal and regulatory issues governing cannabis and cannabis-derived products in the United States. Front Plant Sci. (2019) 10:697. 10.3389/fpls.2019.00697 31263468 PMC6590107

[12] Brutlag A Hommerding H. Toxicology of marijuana, synthetic cannabinoids, and cannabidiol in dogs and cats. Vet Clin North Am Small Anim Pract. (2018) 48:1087–102. 10.1016/j.cvsm.2018.07.008 30342565

[13] Belyea DA Alhabshan R. del Rio-Gonzalez AM, Chadha N, Lamba T, Golshani C, et al. Marijuana use among patients with glaucoma in a city with legalized medical marijuana use. JAMA ophthalmol. (2016) 134:259–64. 10.1001/jamaophthalmol.2015.5209 26719907

[14] Mousa A Petrovic M Fleshner NE. Prevalence and predictors of cannabis use among men receiving androgen-deprivation therapy for advanced prostate cancer. Can Urol Assoc J. (2020) 14:20–6. 10.5489/cuaj.5911 31658007 PMC6955174

[15] Walsh Z Callaway R Belle-Isle L Capler R Kay R Lucas P et al. Cannabis for therapeutic purposes: patient characteristics, access, and reasons for use. Int J Drug Policy. (2013) 24:511–6. 10.1016/j.drugpo.2013.08.010 24095000

[16] Suraev AS Todd L Bowen MT Allsop DJ McGregor IS Ireland C et al. An Australian nationwide survey on medicinal cannabis use for epilepsy: history of antiepileptic drug treatment predicts medicinal cannabis use. Epilepsy Behav. (2017) 70:334–40. 10.1016/j.yebeh.2017.02.005 28238865

[17] Belendiuk KA Baldini LL Bonn-Miller MO. Narrative review of the safety and efficacy of marijuana for the treatment of commonly state-approved medical and psychiatric disorders. Addict Sci Clin Pract. (2015) 10:10. 10.1186/s13722-015-0032-7 25896576 PMC4636852

[18] Kruger DJ Kruger JS. Medical cannabis users' comparisons between medical cannabis and mainstream medicine. J Psychoactive Drugs. (2019) 51:31–6. 10.1080/02791072.2018.1563314 30616501

[19] Porter BE Jacobson C. Report of a parent survey of cannabidiol-enriched cannabis use in pediatric treatment-resistant epilepsy. Epilepsy Behav. (2013) 29:574–7. 10.1016/j.yebeh.2013.08.037 24237632 PMC4157067

[20] Wang GS. Pediatric concerns due to expanded cannabis use: unintended consequences of legalization. J Med Toxicol. (2017) 13:99–105. 10.1007/s13181-016-0552-x 27139708 PMC5330955

[21] Kogan LR Hellyer PW Robinson NG. Consumers' perceptions of hemp products for animals. AHVMA J. (2016) 42:40–8.

[22] Kogan LR Hellyer PW Schoenfeld-Tacher R. Dog owners' use and perceptions of cannabis products. AHVMA J. (2018) 51:26–33. 31281193 PMC6563876

[23] Kogan LR Hellyer PW Silcox S Schoenfeld-Tacher R. Canadian dog owners' use and perceptions of cannabis products. Can Vet J. (2019) 60:749–55. 31281193 PMC6563876

[24] Official Gazette of the Republic of Slovenia. Regulation on the classification of illicit drugs. (2019) Available online at: https://www.uradni-list.si/glasilo-uradni-list-rs/vsebina/2019-01-3115/uredba-o-razvrstitvi-prepovedanih-drog (accessed July 1, 2021).

[25] Bagar T Nolimal D. ICANNA: cannabis in Slovenia. London: Medical cannabis network. (2020) Available online at: https://www.healtheuropa.eu/icanna-cannabis-in-slovenia/102610/ (accessed June 23, 2021).

[26] Reid MA. qualitative review of cannabis stigmas at the twilight of prohibition. J Cannabis Res. (2020) 2:46. 10.1186/s42238-020-00056-8 33526147 PMC7819345

[27] European, Commission,. Novel Food Catalogue. Available online at: https://ec.europa.eu/food/safety/novel_food/catalogue/search/public/index.cfm (accessed 23.6.2021).

[28] Corroon J Phillips JAA. cross-sectional study of cannabidiol users. Cannabis Cannabinoid Res. (2018) 3:152–61. 10.1089/can.2018.0006 30014038 PMC6043845

[29] Leas EC Hendrickson EM Nobles AL. Self-reported cannabidiol (CBD) use for conditions with proven therapies. JAMA Netw Open. (2020) 3:e2020977. 10.1001/jamanetworkopen.2020.20977 33057645 PMC7563067

[30] Moltke J Hindocha C. Reasons for cannabidiol use: a cross-sectional study of CBD users, focusing on self-perceived stress, anxiety, and sleep problems. J Cannabis Res. (2021) 3:5. 10.1186/s42238-021-00061-5 33602344 PMC7893882

[31] Philpot LM Ebbert JO Hurt RTA. survey of the attitudes, beliefs and knowledge about medical cannabis among primary care providers. BMC Fam Pract. (2019) 20:17. 10.1186/s12875-019-0906-y 30669979 PMC6341534

[32] Carlini BH Garrett SB Carter GT. Medicinal cannabis: a survey among health care providers in Washington state. Am J Hosp Palliat Care. (2017) 34:85–91. 10.1177/1049909115604669 26377551

[33] Gamble LJ Boesch JM Frye CW Schwark WS Mann S Wolfe L et al. Pharmacokinetics, safety, and clinical efficacy of cannabidiol treatment in osteoarthritic dogs. Front Vet Sci. (2018) 5:165. 10.3389/fvets.2018.00165 30083539 PMC6065210

[34] McGrath S Bartner LR Rao S Packer RA Gustafson DL. Randomized blinded controlled clinical trial to assess the effect of oral cannabidiol administration in addition to conventional antiepileptic treatment on seizure frequency in dogs with intractable idiopathic epilepsy. J Am Vet Med Assoc. (2019) 254:1301–8. 10.2460/javma.254.11.1301 31067185

[35] Morris EM Kitts-Morgan SE Spangler DM McLeod KR Costa JHC Harmon DL. The impact of feeding cannabidiol (CBD) containing treats on canine response to a noise-induced fear response test. Front Vet Sci. (2020) 7:569565. 10.3389/fvets.2020.569565 33195551 PMC7537661

[36] Deabold KA Schwark WS Wolf L Wakshlag JJ. Single-Dose Pharmacokinetics and Preliminary Safety Assessment with Use of CBD-Rich Hemp Nutraceutical in Healthy Dogs and Cats. Animals (Basel). (2019) 9:832. 10.3390/ani9100832 31635105 PMC6826847

[37] Kogan L Hellyer P Downing R. The use of cannabidiol-rich hemp oil extract to treat canine osteoarthritis-related pain: A pilot study. AHVMA J. (2020) 58:1−10.

[38] Mcgrath S Bartner LR Rao S Kogan LR Hellyer PWA. report of adverse effects associated with the administration of cannabidiol in healthy dogs. AHVMA J. (2018) 52:34–8.

[39] Vaughn D Kulpa J Paulionis L. Preliminary investigation of the safety of escalating cannabinoid doses in healthy dogs. Front Vet Sci. (2020) 7:e51. 10.3389/fvets.2020.00051 32118071 PMC7029731

[40] Wakshlag JJ Schwark WS Deabold KA Talsma BN Cital S Lyubimov A. Pharmacokinetics of cannabidiol, cannabidiolic acid, Δ9-Tetrahydrocannabinol, tetrahydrocannabinolic acid and related metabolites in canine serum after dosing with three oral forms of hemp extract. Front Vet Sci. (2020) 7:e505. 10.3389/fvets.2020.00505 33102539 PMC7498943

[41] Payre W Cestac J Delhomme P. Intention to use a fully automated car: attitudes and a priori acceptability. Transp Res F: Traffic Psychol Behav. (2014) 27:252–63. 10.1016/j.trf.2014.04.009

[42] Vogel EA Ramo DE Rubinstein ML Delucchi KL Darrow SM Costello C et al. Effects of social media on adolescents' willingness and intention to use e-cigarettes: an experimental investigation. Nicotine Tob Res. (2021) 23:694–701. 10.1093/ntr/ntaa003 31912147 PMC7976937

[43] D'Amico EJ Miles JN Tucker JS. Gateway to curiosity: Medical marijuana ads and intention and use during middle school. Psychol Addict Behav. (2015) 29:613–9. 10.1037/adb0000094 26030167 PMC4587352

[44] Liébana-Presa C Martínez-Fernández MC Benítez-Andrades JA Fernández-Martínez E Marqués-Sánchez P García-Rodríguez I. Stress, emotional intelligence and the intention to use cannabis in Spanish adolescents: influence of COVID-19 confinement. Front Psychol. (2020) 11:582578. 10.3389/fpsyg.2020.582578 33362646 PMC7759484

[45] Premik M. Alternative medicine in Slovenia: some social-medical views. Health Care Anal:. (1998) 6:59–64. 10.1002/(SICI)1099-1042(199803)6:1&lt 10179298

[46] Eisenberg DM Kessler RC Foster C Norlock FE Calkins DR Delbanco TL. Unconventional medicine in the United States. prevalence, costs, and patterns of use. N Engl J Med. (1993) 328:246–52. 10.1056/NEJM199301283280406 8418405

[47] Kelner M Wellman B. Health care and consumer choice: medical and complementary therapies. Soc Sci Med. (1997) 45:203–12. 10.1016/S0277-9536(96)00334-6 9225408

